# Comparative assessment of pharmacological and nonpharmacological anxiety management in pediatric dentistry - An *in vivo* study

**DOI:** 10.6026/973206300212044

**Published:** 2025-07-31

**Authors:** Udita Samanta, Ashjan Ashraf Batha, Nitin K. Som, Sunny Yadav, Shipra Jaidka, Deepti Jawa

**Affiliations:** 1 Department of Paediatric and Preventive Dentistry, Divya Jyoti College of Dental Science and Research, Modinagar, Uttar Pradesh 201204, India

**Keywords:** Anxiety, nitrous oxide, midazolam, emotional freedom technique, sphygmomanometer, sedation

## Abstract

The effectiveness of pharmacological and non-pharmacological anxiety management techniques in pediatric patients is of interest. Sixty children (5-12 years) with Frankl negative behavior requiring local anesthesia for extraction were divided into four groups: Group 1 (N_2_O sedation), Group 2 (oral midazolam with fruit juice), Group 3 (EFT), and Group 4 (no sedation). Anxiety levels (VAS), oxygen saturation, pulse rate (pulse oximeter), and blood pressure (sphygmomanometer) were measured. N_2_O sedation proved most effective, with EFT being a promising non-pharmacological alternative. Combining both techniques can optimize pediatric patient management.

## Background:

Dental anxiety remains a significant barrier to effective oral health care in children, often rooted in fear of pain, needles, past traumatic experiences, and a general fear of the unknown. Defined as an emotional reaction to perceived dental threats, dental anxiety can manifest as discomfort, nervousness, or even panic, especially during or before dental procedures [1]. If unaddressed, it can lead to avoidance of dental care, negatively impacting a child's emotional well-being and oral health. The causes of dental anxiety in children are multifactorial and may include both psychological and physiological components [2]. Over the years, a wide range of behavioural management techniques have been developed, from conventional non-pharmacological methods like distraction, cognitive-behavioural therapy, and physical restraint to advanced pharmacological sedation options [3]. Nowadays, many approaches have also explored integrative methods that address emotional energy and psychological stress. One of a kind technique is the Emotional Freedom Technique (EFT), which is an emerging non-pharmacological method derived from traditional Chinese treatment [4]. It involves tapping on specific acupressure points to balance the body's energy system and release negative emotions, eventually reducing anxiety. It is a non-invasive, quick, and self-administered technique, particularly beneficial for children who respond well to mind and body approaches. EFT has shown promise in promoting emotional regulation and relaxation without the involvement of medication [5]. On the other hand, nitrous oxide inhalation sedation and oral midazolam remain widely used and effective options. Nitrous oxide was introduced in dentistry by Dr. Horace Wells in 1844 [6]. It offers a safe and adjustable sedation method that allows children to remain conscious and cooperative during treatment. Its rapid onset and recovery, combined with minimal side effects, make it ideal for mild to moderate anxiety management [7]. For more uncooperative patients, oral midazolam, which is a benzodiazepine with anxiolytic, sedative and amnesic properties, is preferred due to its ease of administration and effectiveness, especially in highly anxious or uncooperative children [8, 9]. As the diversity of responses in paediatric patients varies to a large extent, sometimes a combination of behavioural and pharmacological techniques is employed to ensure optimal comfort and cooperation. Therefore, it is of interest to evaluate and compare the effectiveness of newer anxiety management strategies, like Emotional Freedom Technique, with conventional nitrous oxide sedation and oral midazolam sedation to reduce dental anxiety among children. The intention is to create a positive, stress-free dental environment that encourages regular dental visits and fosters long-term oral health.

## Materials and Methods:

For this study, 60 children within the age group of 5-12 years [10] showing Frankel behaviour rating 1 and 2 were selected.

## Inclusion criteria:

[1] Children in the age group 5-12 years

[2] Children who required extraction under local anesthesia

[3] Children showing 1 and 2 behaviours on Frankl's rating scale

## Exclusion criteria:

[1] Children without consent from their parents or guardians

[2] Physically Challenged children

[3] Mentally Challenged children

The study assessed the efficiency and efficacy of sedation agents in anxiety management. Efficiency was determined by measuring the onset time using a digital stopwatch, while efficacy was evaluated through pulse rate, oxygen saturation (SpO_2_), blood pressure, and anxiety scores on the Visual Analogue Scale (VAS). In the Nitrous Oxide group, patients followed strict pre-operative dietary restrictions and underwent nasal patency checks. Sedation was induced with incremental nitrous oxide administration following oxygen pre-oxygenation, monitored using Ramsay Sedation Score, SpO_2_ and pulse rate. Local anesthesia was followed by optimal sedation. Post-procedure, nitrous oxide was withdrawn and oxygen administered; recovery was assessed via walking and a puzzle game. The Oral Midazolam group also observed dietary restrictions; dosage (0.5 mg/kg) was calculated by body weight and administered in juice. Vitals and anxiety levels were recorded; flumazenil was kept ready as a reversal agent. All sedation durations were recorded digitally. Sedation onset was recorded using a stopwatch. Patients remained seated for 10-15 minutes, with sedation depth assessed using the Ramsay Sedation Score, confirming signs like drowsiness and reduced pain response before administering local anesthesia. In the post-operative phase, patients were monitored until reaching Ramsay Sedation Score 2. Water intake was encouraged, and additional medications were prohibited for 24 hours. Follow-up was scheduled within 2-3 days, with hourly telephonic monitoring until full recovery. Vital signs, anxiety levels, and sedation time were recorded. EFT patients underwent procedural briefing with no pre-operative restrictions. Anxiety was recorded using VAS, while vital signs were monitored. Patients identified their fear, rated it, and performed EFT tapping on meridian points under guidance. The process was repeated until anxiety was reduced by five points. Local anesthesia was then administered, and dental extraction occurred. Additional EFT cycles were used if necessary. Post-operatively, patients were discharged with medications and care instructions. Vital signs, anxiety scores, and sedation time were recorded. The control group received no anxiety management. Routine clinical monitoring was performed.

## Results:

N_2_O significantly reduced anxiety (57.6%), followed by EFT (46.53%) and Oral Midazolam (42.04%), while no sedation increased anxiety. One-way ANOVA confirmed statistically significant differences (p = 0.001) (Table 1 - see PDF and Figure 1 - see PDF). N_2_O showed the highest pulse rate reduction (7.31%), followed by EFT (4.67%) and Oral Midazolam (4.08%); whereas no sedation group increased pulse rate (Table 2 - see PDF). N_2_O significantly increased oxygen levels; Oral Midazolam decreased them, while EFT and no sedation showed minimal, non-significant changes (Table 3 - see PDF). Intragroup comparison showed significant changes in DBP (p = 0.001), with N_2_O reducing Diastolic blood pressure most, followed by Oral Midazolam, EFT, and a rise in the No Sedation group (Table 4 - see PDF). Intragroup comparison showed significant Systolic blood pressure changes (p = 0.001), with N_2_O reducing SBP most, followed by EFT, Oral Midazolam, and a rise in the No Sedation group (Table 5 - see PDF). Sedation time varied significantly (p = 0.001), with N_2_O being the fastest (14.87 min), followed by Oral Midazolam (25.13 min), and EFT the longest (34.67 min) (Table 6 - see PDF and Figure 2 - see PDF).

## Discussion:

Dental anxiety is a prevalent psychological condition, particularly among children, where fear and apprehension towards dental procedures significantly compromise oral health. Deva *et al.* (2016) [9] observed that children with dental anxiety often avoid dental visits, which results in delayed treatment and poor oral hygiene. Around 20% of children experience this anxiety driven by fear of pain, unfamiliar clinical settings, and previous negative dental encounters. As reported by Shehani *et al.* [10], non-pharmacological approaches are generally preferred as first-line interventions. Conventional techniques such as Tell-Show-Do (TSD), modeling and distraction therapy have shown a significant effect. According to Browder *et al.* (2012) [11], TSD involves explaining and demonstrating procedures in a child-friendly manner and enhances cooperation to reduce fear. Similarly, Nada Farhat-McHayleh *et al.* (2009) [12] demonstrated the efficacy of modeling, where children observe peers undergoing dental procedures, thereby normalizing the experience. Distraction methods like audiovisual therapy were highlighted by Khandelwal *et al.* (2019) [13] as effective in diverting the child's attention. Areef *et al.* (2024) [14] introduced alpha wave music to calm anxious children. Positive reinforcement has also proved valuable in accordance with Shehani *et al.* (2024) [10], noting its role in promoting compliance and reducing dental-related fear. Sometimes, psychological interventions are not enough pharmacological methods such as oral sedation, nitrous oxide inhalation, and general anesthesia are employed. Rao and Tiwari (2024) [15] identified midazolam as a preferred sedative option for children due to its rapid onset of action with fewer adverse effects. Nitrous oxide (N_2_O), as noted Devi & Jeevanandan *et al.* (2024) [16], is popular for its quick recovery time. Innovative approaches like hypnosis, the Magnetic Finger Method, and Emotional Freedom Technique (EFT) are also emerging. Temple *et al.* (2011) [17] explained that the Magnetic Finger Method, by using guided imagery and tactile stimulation, effectively reduces anxiety. Shehani *et al.* (2024) [10] explored EFT by involving acupressure tapping on meridian points to alleviate stress and anxiety. While these methods show future potential, but still require further validation. In this study, 60 paediatric patients aged 5-12 were involved because dental anxiety is most pronounced at this age in accordance with Farzanegan *et al.* (2025) [18]. Extractions were selected as the standard procedure to assess anxiety due to their higher stress induction compared to restorative treatments, as suggested by Prado *et al.* (2024) [19]. The Visual Analogue Scale (VAS) was used for assessment, favoured for its reliability and simplicity as reported by Shehani *et al.* (2024) [10], while physiological monitoring was conducted via digital pulse oximetry to ensure precision highlighted by Sukumaran *et al.* (2025) [20]. Statistical analysis revealed nitrous oxide sedation as the most effective intervention, significantly reducing anxiety with a faster onset. Wu *et al.* (2025) [21] explained that N_2_O modulates GABA receptors and inhibits NMDA receptors, producing calming effects without impairing reflexes. Physiological responses included reduced pulse and blood pressure, indicating lower sympathetic activity Kanagasundaram *et al.*, 2001) [22]. Comparatively, EFT performed similarly to midazolam in reducing anxiety, functioning through cognitive restructuring and modulation of cortisol and amygdala activity Bach *et al.*, 2003) [23]. Midazolam, though fast-acting, showed limitations due to its taste and side effects Misaka *et al.* 2010) [24]. In the absence of anxiety control, children displayed heightened stress, reflected in increased VAS scores and physiological parameters, aligning with findings by Achmad *et al.* (2019) [25]. These outcomes underscore the necessity of employing effective, child-centered anxiety management strategies in dental care.

## Conclusion:

N_2_O inhalation sedation was the most effective in reducing pulse rate, blood pressure, and anxiety levels, followed by the emotional freedom technique (EFT), with oral midazolam showing the least reduction. Oxygen saturation remained stable with EFT but slightly decreased with oral sedation. Anxiety and physiological parameters significantly increased without intervention. EFT is a safe, economical, and non-pharmacological alternative for managing dental anxiety in children.
